# Commentary: Soluble CD83 Alleviates Experimental Autoimmune Uveitis by Inhibiting Filamentous Actin-Dependent Calcium Release in Dendritic Cells

**DOI:** 10.3389/fimmu.2018.02659

**Published:** 2018-11-15

**Authors:** Mariana Pereira Pinho, José Alexandre Marzagão Barbuto

**Affiliations:** Tumor Immunology Laboratory, Immunology Department, Biomedical Sciences Institute, University of São Paulo, São Paulo, Brazil

**Keywords:** CD83, calcium signaling, dendritic cells, T lymphocytes, T cell activation

CD83 is a molecule known as an activation marker of dendritic cells (DC), that exists in two different forms—membrane-bound and soluble—which have antagonistic immunomodulatory functions (1). The soluble form (sCD83) consist of the extracellular domain of membrane CD83 (mCD83), and has immunosuppressive functions (2, 3). Its potential to be used in the treatment of autoimmune disease has been frequently assessed (4, 5). In the recent issue of Frontiers in Immunology, Lin et al. (6) consistently showed the positive effect of the use of sCD83 to treat experimental autoimmune uveitis.

The authors went one step further and described the mechanism of action by which the soluble form of CD83 was able to protect against the uveitis in mice. They showed that sCD83-treated DC, as well as T cells co-cultured with these cells, had lower intracellular calcium signaling, an effect that was dose-dependent. This is the second report showing that CD83 molecules can interfere with calcium signaling. We had previously shown that the membrane form of CD83, present in the surface of mature DC, was important to induce higher calcium signaling on the T cells (7). Thus, both studies elegantly support the opposed functions of soluble and membrane-bound CD83, with lower, in the presence of sCD83, vs. higher calcium signaling induced by mCD83.

The elevation of intracellular calcium usually requires two steps. The first starts with the activation of phospholipase C (PLCγ) that produces inositol 1,4,5-trisphosphate (IP3) which, in turn, binds to its receptor in the membrane of the endoplasmic reticulum (ER), causing the release of calcium stored in the ER. The second step is the amplification phase, where the depletion of ER calcium stores is sensed by STIM1, leading to the opening of CRAC (calcium release–activated calcium) channels, composed of ORAI subunits, in the plasma membrane of the cell. This causes a sustained elevation of the intracellular calcium concentration that will lead to cell activation (8). The article showed that sCD83 treatment prevents the co-localization of ORAI1 and mitochondria, two important pieces to sustain the calcium signaling (9), in the immunological synapsis of DC and T cells. Thus, sCD83 would act interfering with the calcium influx, the second step in the calcium signaling. On the other hand, our study suggests that mCD83 acts in the first phase of calcium signaling. We showed that, in the absence of extracellular calcium, blockage of mCD83 seemed to abrogate the residual increase in the intracellular calcium concentration, indicating that mCD83 was important to generate the initial release of calcium from the ER.

These data seem to be contradictory, but can be explained by the last piece of evidence showed by Lin and co-authors. They showed that sCD83 interferes with F-actin accumulation in the immunological synapsis, which was responsible for the aberrant localization of ORAI1 in sCD83-treated DC. The ability of sCD83 to modulate DC cytoskeleton had already been reported before (10). In our work, we hypothesized that CD83 was responsible for bringing the complex comprising both LAT and PLCγ closer to the TCR, thus boosting the first phase of calcium signaling. Although not fully established, this co-localization seems to require cytoskeletal rearrangement (11). Thus, by interfering with actin localization, mCD83 molecules could boost both the first and second step of calcium signaling. If CD83-induced actin-relocation is dependent of its interaction with a ligand, sCD83, when binding to CD83 ligand, would prevent this signaling, similarly to the effect of a blocking antibody, leading to diminished calcium signaling.

Altogether, these findings help to elucidate the mechanism by which CD83 molecules exert their immunomodulatory functions, generating relevant information that can be used to improve and refine the strategies aiming to interfere with this axis.

## Author contributions

All authors listed have made a substantial, direct and intellectual contribution to the work, and approved it for publication.

### Conflict of interest statement

The authors declare that the research was conducted in the absence of any commercial or financial relationships that could be construed as a potential conflict of interest.
